# Clinical consequences of consecutive self-expanding transcatheter heart valve iterations

**DOI:** 10.1007/s12471-021-01568-5

**Published:** 2021-04-29

**Authors:** H. G. Kroon, L. van Gils, F. Ziviello, M. P. H. van Wiechen, J. F. W. Ooms, Z. Rahhab, N. El Faquir, A.‑M. Maugenest, J. A. Goudzwaard, P. Cummins, M. Lenzen, I. Kardys, J. Daemen, F. Mattace-Raso, P. P. T. de Jaegere, N. M. Van Mieghem

**Affiliations:** Department of Cardiology, Thoraxcenter, Erasmus Medical Center, Rotterdam, The Netherlands

**Keywords:** Transcatheter valve interventions, Aortic stenosis

## Abstract

**Objective:**

To compare early clinical outcomes after transcatheter aortic valve implantation (TAVI) with three consecutive generations of self-expanding valves (SEVs).

**Methods:**

Clinical endpoints of consecutive patients who underwent TAVI with CoreValve, Evolut R or Evolut PRO were included in a prospective database.

**Results:**

TAVI was performed with CoreValve (*n* = 116), Evolut R (*n* = 160) or Evolut PRO (*n* = 92). Evolut R and Evolut PRO showed a tendency towards lower permanent pacemaker implantation (PPI) rates compared to CoreValve (CoreValve 27% vs Evolut R 16% vs Evolut PRO 18%, *p* = 0.091). By multivariable regression analysis CoreValve had a significantly higher risk for PPI (odds ratio (OR) 2.79, 95% confidence interval (CI) 1.31–5.94, *p* = 0.008) compared to Evolut R, while Evolut R and PRO were similar. Severe paravalvular leakage (PVL) occurred only with CoreValve, but no significant difference was observed in moderate PVL (10% vs 8% vs 6%, *p* = 0.49). CoreValve had a tendency towards a higher risk for more-than-mild PVL as compared with the Evolut platform (R + PRO) (OR 2.46, 95% CI 0.98–6.16, *p* = 0.055). No significant differences in all-cause mortality (7% vs 4% vs 1%, *p* = 0.10), stroke (6% vs 3% vs 2%, *p* = 0.21) or major vascular complications (10% vs 12% vs 4%, *p* = 0.14) were observed.

**Conclusions:**

TAVI with self-expanding valves was safe, and device iterations may result in a lower need for PPI. More-than-mild PVL seemed to occur less often with repositionable technology.

**Supplementary Information:**

The online version of this article (10.1007/s12471-021-01568-5) contains supplementary material, which is available to authorized users.

## What’s new?

- Transcatheter aortic valve implantation using Evolut R and Evolut PRO seemed to be associated with a lower need for permanent pacemaker implantations compared to CoreValve.
- More-than-mild paravalvular leakage seemed to occur less often with repositionable technology.
- Addition of a sealing wrap in Evolut PRO did not further reduce the risk for more-than-mild paravalvular leakage compared to Evolut R in this study.

## Introduction

Transcatheter aortic valve implantation (TAVI) with the CoreValve transcatheter heart valve (THV) (Medtronic Inc., Minneapolis, MN, USA) demonstrated superior haemodynamic valve performance compared to surgery but resulted in more paravalvular leakage (PVL) and permanent pacemaker implantations (PPIs) [1]. The second-generation Evolut R has a smaller profile size and can be recaptured/repositioned to optimise device positioning and thereby limit both PVL and high-degree atrioventricular blocks [2]. Three large registries reported a 14.7–19.3% PPI rate and a 1.9–7.7% rate of more-than-mild PVL [2–4]. The third-generation Evolut PRO features a pericardial wrap to further reduce PVL [5]. The US Evolut PRO Registry included 60 patients. More-than-mild PVL did not occur and 12% of patients required a PPI [5]. Both PPI and more-than-mild PVL are associated with worse clinical outcomes after TAVI [6, 7]. In this study we aimed to compare the early clinical performance of these three consecutive self-expanding valves (SEVs) in a real-world TAVI population.

## Methods

### Patient selection

All patients who underwent transfemoral or transsubclavian TAVI with one of the three SEVs for severe aortic stenosis (AS) between January 2012 and December 2018 were entered into our prospective database. Patients with a pacemaker at baseline were excluded (*n* = 37). All patients provided written informed consent for the TAVI procedure and subsequent data analysis. This present study was in accordance with the Declaration of Helsinki and approved by our center’s Institutional Review Board.

### Primary and secondary outcomes

The primary outcomes for this study were: (1) the need for PPI at 30 days, (2) more-than-mild PVL on pre-discharge transthoracic echocardiographic evaluation based on Valve Academic Research Consortium (VARC)-2 criteria [8]. Whether or not a patient required PPI was the treating physician’s decision but in general in compliance with the ESC guidelines on cardiac pacing and resynchronisation therapy. Secondary endpoints were in-hospital stroke, vascular and bleeding complications and 30-day all-cause mortality according to VARC‑2 criteria [8]. All treated patients were discussed in our weekly plenary session with the interventionalists and came to our outpatient clinic at 30 days for adjudication of all events.

### Data analysis

We defined the sheath to femoral artery ratio (SFAR) as the ratio between the sheath outer diameter (in millimetres) and the femoral artery minimal lumen diameter (in millimetres). The annular sizing ratio was defined as the ratio between the labelled prosthesis diameter and the perimeter-derived annular diameter. Continuous variables were presented as mean (±SD) or median (interquartile range, IQR) and categorical variables as *n* (%). The distribution of continuous variables was examined for normality through histograms and Q‑Q plots. For the comparison of continuous variables between the three different THV’s one-way ANOVA or the non-parametric Kruskal-Wallis test was performed, according to the distribution of the variables. For the comparison of categorical variables the Pearson χ^2^ or Fisher exact test was used as appropriate. For ensuing pairwise comparisons, we applied Bonferroni corrections to account for multiple testing. Additionally, we performed multivariable logistic regression to estimate the effect of the three THV’s on the need for PPI at 30 days. We entered THV type and added baseline covariates that displayed a difference with a *p*-value less than 0.10 in univariate analysis. Also, when the number of events was sufficient, we added baseline variables that were different among the three valve types. To avoid over-adjustment, we chose to exclude the repositioning feature in this logistic analysis, because this is intrinsic to the Evolut design. In a second analysis we estimated the effect of the three THV’s on the occurrence of more-than-mild PVL, together with baseline covariates selected as described previously. If there was a limited number of events we chose those covariates that had a *p*-value less than 0.10 and that are known (or theoretical) risk factors for the occurrence of PVL (annular anatomical features, calcification levels of left ventricular outflow tract (LVOT) or annulus). Lastly, we combined all Evolut R and PRO patients and compared this group to the CoreValve patients, as the newer generations were built on more or less the same fundamentals. All statistical analyses were performed with SPSS version 24.0 (IBM Corporation, Armonk, NY, USA). A two-sided value of *p* < 0.05 was considered statistically significant.

## Results

A total of 368 (53% male) patients underwent TAVI with CoreValve (*n* = 116), Evolut R (*n* = 160) or Evolut PRO (*n* = 92). The overall median age and Society of Thoracic Surgeons (STS) predicted risk of mortality were 80 (IQR 74–84) years and 4.3% (IQR 2.8–6.3), respectively. TAVI was performed via the transfemoral route in 89% of cases. No significant differences were observed regarding moderate or severe LVOT and annular calcifications or the presence of (functional) bicuspid valves. All baseline characteristics are shown in Tab. 1.

**Table 1:** Baseline characteristics of patients and procedural details for the three self-expanding valves

	CoreValve (*n* = 116)	Evolut R (*n* = 160)	Evolut PRO (*n* = 92)	*p*-value
*Baseline characteristics*				
Male gender	68 (59%)	86 (54%)	42 (46%)	0.17
Age (years)	80 (75–84)	79 (73–84)	80 (74–85)	0.32
STS score (%)	4.3 (3.1–5.7)	4.2 (2.7–6.4)	4.3 (2.5–6.5)	0.86
Creatinine at baseline (µmol/l)	95 (75–128)	98 (75–117)	90 (74–117)	0.56
Body mass index (kg/m^2^)	27 ± 4	27 ± 5	27 ± 5	0.78
Ischaemic heart disease	53 (46%)	70 (44%)	31 (34%)	0.18
History of AVR/TAVI	5 (4%)	14 (9%)	7 (8%)	0.36
Diabetes mellitus	38 (33%)	44 (28%)	29 (32%)	0.66
Hypertension	87 (75%)	121 (76%)	71 (77%)	0.93
History of atrial fibrillation	28 (24%)	47 (29%)	22 (24%)	0.52
History of stroke	21 (18%)	18 (11%)	14 (15%)	0.27
Peripheral arterial disease	43 (37%)	80 (50%)^c^	27 (29%)^c^	* 0.003*
NYHA class ≥3	84 (75%)^a^	93 (58%)^a^	62 (68%)	* 0.035*
Bicuspid valve (functional)	9 (8%)	10 (6%)	7 (8%)	0.92
Moderate or severe annulus calcification (Rosenhek)	92 (79%)	126 (79%)	77 (84%)	0.46
Moderate or severe LVOT calcification	20 (17%)	22 (14%)	14 (15%)	0.65
*Baseline conduction disturbances (alone or in combination)*				
RBBB	7 (6%)	15 (10%)	11 (12%)	0.32
LBBB	18 (16%)	11 (7%)	6 (7%)	* 0.03*
UIVD	3 (3%)	3 (2%)	3 (3%)	0.79
AV1B	26 (22%)	28 (18%)	21 (23%)	0.52
LAFB	15 (13%)	12 (8%)	4 (4%)	0.08
LPFB	1 (1%)	1 (1%)	0 (0%)	0.69
*Procedural details*				
Femoral access	97 (84%)^b^	142 (89%)	89 (97%)^b^	* 0.01*
Target access vessel diameter (mm)	7.0 ± 1.6	6.7 ± 1.4^c^	7.4 ± 1.3^c^	* 0.002*
Sheath to femoral artery ratio	1.03 (0.87–1.23)^a,b^	0.87 (0.75–0.97)^a^	0.80 (0.72–0.93)^b^	*<0.005*
Valve sizes (mm)				**–**
23	2 (2%)	7 (4%)	2 (2%)	
26	27 (23%)	49 (31%)	30 (33%)	
29	58 (50%)	78 (49%)	60 (65%)	
31	29 (25%)	–	–	
34	–	26 (16%)	–	
Perimeter derived annular diameter (mm)	25.0 (23.4–26.7)^a,b^	23.9 (22.4–25.5)^a^	23.8 (22.5–24.9)^b^	*<0.005*
Annular sizing ratio	1.15 (1.10–1.20)^a,b^	1.19 (1.15–1.23)^a^	1.18 (1.14–1.21)^b^	*<0.005*
Depth of implantation (mm)	7.0 ± 3.2	7.0 ± 3.3	7.2 ± 2.6	0.85
Pre-dilatation	92 (79%)^a,b^	12 (8%)^a^	11 (12%)^b^	*<0.005*
Post-dilatation	32 (28%)^a^	67 (42%)^a^	38 (41%)	* 0.03*
Repositioning used?	–	56 (35%)	17 (19%)	*<0.005*
One time		35	6	
Two times		14	7	
Three times		4	2	
Four times		3	2	
Valve in valve during procedure	8 (7%)	6 (4%)	4 (4%)	0.47
Number of valves implanted	1, range 1–3	1, range 1–3	1, range 1–2	0.23

Categorical variables are shown as *n* (%). Continuous variables are displayed as mean ± SD, median (interquartile range) or median, range

*STS* Society of Thoracic Surgeons, *AVR* aortic valve replacement, *TAVI* transcatheter aortic valve implantation, *NYHA* New York Heart Association, *RBBB* right bundle branch block, *LBBB* left bundle branch block, *UIVD* unspecific intraventricular conduction defect, *AV1B* first-degree atrioventricular block, *LAFB* left anterior fascicular block, *LPFB* left posterior fascicular block

^a, b, c^*p* < 0.05 for pairwise comparisons with Bonferroni correction

### Clinical outcomes among the three generations of SEV

TAVI with Evolut R and Evolut PRO was performed more often via a femoral approach (CoreValve 84% vs Evolut R 89% vs Evolut PRO 97%, *p* = 0.01), without pre-dilatation (79% vs 8% vs 12%, *p* < 0.005) and with post-dilatation (28% vs 42% vs 41%, *p* = 0.03). Repositioning was done twice as often with the Evolut R as with the Evolut PRO valve (35% vs 19%, *p* < 0.005). The SFAR was lower with the two newer generations, while the annular sizing ratio was higher. There was a lower PPI rate with Evolut R and Evolut PRO than with the CoreValve THV (27% vs 16% vs 18%, *p* = 0.091; see Tab. 2 and Electronic Supplementary Material: Fig. S1). Multivariable logistic regression analysis showed CoreValve TAVI to result in a higher need for PPI compared with Evolut R, but there was no difference between Evolut R and Evolut PRO (odds ratio (OR) 2.79, 95% confidence interval (CI) 1.31–5.94, *p* = 0.008 and OR 1.18, 95% CI 0.52–2.71, *p* = 0.69 respectively; Table 3). The univariate analyses of the pre-specified clinical endpoints can be found in the Electronic Supplementary Material (Tables S1 and S2). The incidence of PPI after CoreValve TAVI was higher than for the Evolut platform combined (R + PRO) (OR 2.56, 95% CI 1.29–5.08, *p* = 0.007), as displayed in the Electronic Supplementary Material (Tables S3 and S4). Right bundle branch block (RBBB), first-degree atrioventricular block at baseline and deeper implantations were independent predictors for PPI.

**Table 2:** Clinical outcomes with Medtronic CoreValve, Evolut R and Evolut PRO

	CoreValve (*n* = 116)	Evolut R (*n* = 160)	Evolut PRO (*n* = 92)	*p*-value
*Clinical outcomes*				
Permanent pacemaker post-TAVI owing to:	31 (27%)	26 (16%)	17 (18%)	0.091
AV3B	20 (65%)	23 (89%)	14 (82%)	–
AV2B (Mobitz II)	3 (10%)	1 (4%)	3 (18%)	–
AF with bradycardia	3 (10%)	0 (0%)	0 (0%)	–
Sick sinus syndrome	3 (10%)	0 (0%)	0 (0%)	–
Other	2 (7%)	2 (8%)	0 (0%)	–
Mortality at 30 days	8 (7%)	6 (4%)	1 (1%)	0.10
Moderate to severe paravalvular leakage	15 (13%)	12 (8%)	5 (6%)	0.11
Moderate	11 (10%)	12 (8%)	5 (6%)	
Severe	4 (3%)	0 (0%)	0 (0%)	
Minor vascular complication	11 (10%)	11 (7%)	3 (3%)	0.21
Major vascular complication	12 (10%)	19 (12%)	4 (4%)	0.14
Minor bleeding	7 (6%)	15 (9%)	5 (5%)	0.42
Major or life-threatening bleeding	17 (15%)**	23 (14%)***	4 (4%)**^,^***	*0.034*
Major	8 (7%)	14 (9%)	3 (3%)	
Life-threatening	9 (8%)	9 (6%)	1 (1%)	
Any stroke	7 (6%)	4 (3%)	2 (2%)	0.21
Disabling	5 (4%)	2 (1%)	1 (1%)	
*New conduction disturbances*				
Procedural AV3B	24 (21%)	29 (18%)	15 (17%)	0.74
Procedural LBBB	65 (56%)	87 (55%)	51 (55%)	0.99
Procedural RBBB	2 (2%)	2 (1%)	0 (0%)	0.48
Temporary LBBB	37 (32%)	40 (25%)	30 (33%)	0.34
Permanent LBBB	35 (30%)	51 (32%)	29 (32%)	0.95
Temporary RBBB	4 (4%)	10 (6%)	2 (2%)	0.26
Permanent RBBB	0 (0%)**	1 (1%)***	6 (7%)**^,^***	*0.001*
Temporary AV2B/AV3B	8 (7%)	10 (6%)	13 (14%)	0.08
Permanent AV2B/AV3B	20 (17%)	20 (13%)	11 (12%)	0.46
AV1B	22 (19%)	29 (18%)	15 (17%)	0.88
AF	16 (14%)*^,^**	8 (5%)*	3 (3%)**	*0.005*

Categorical variables are shown as *n* (%)

*TAVI* transcatheter aortic valve implantation, *AV3B* third-degree atrioventricular block, *AV2B* second-degree atrioventricular block, *AF* atrial fibrillation, *LBBB* left bundle branch block, *RBBB* right bundle branch block, *AV1B* first-degree atrioventricular block

*^,^**^,^***p < 0.05 for pairwise comparisons with Bonferroni correction

**Table 3:** Multivariable regression analysis on the need for a permanent pacemaker and on paravalvular leakage

	Pacemaker implantation OR (95% CI)	*p*-value
Type of THV used		
Evolut R	1.00 (reference)	–
CoreValve	2.79 (1.31–5.94)	** 0.008**
Evolut PRO	1.18 (0.52–2.71)	0.69
Male gender	0.71 (0.36–1.42)	0.33
RBBB at baseline	14.44 (5.59–37.28)	**<0.005**
LAFB at baseline	1.28 (0.46–3.61)	0.64
AV1B at baseline	3.36 (1.64–6.87)	** 0.001**
Mean depth of implantation (mm)	1.21 (1.09–1.34)	**<0.005**
Annular sizing ratio	0.17 (0.002–11.700)	0.41
Peripheral artery disease	1.70 (0.91–3.18)	0.098
	Moderate or severe PVL OR (95% CI)	*p‑value*
Type of THV used		
Evolut R	1.00 (reference)	–
CoreValve	2.06 (0.79–5.39)	0.14
Evolut PRO	0.53 (0.16–1.77)	0.30
Bicuspid valve (functional)	1.51 (0.46–4.96)	0.49
Annular sizing ratio	0.02 (0.000–5.410)	0.18
Post-dilatation	5.56 (2.23–13.86)	**<0.005**

Variables are shown as OR (95% CI)

*OR* odds ratio, *CI* confidence interval, *THV* transcatheter heart valve, *RBBB* right bundle branch block, *LAFB* left anterior fascicular block, *AV1B* first-degree atrioventricular block, *PVL* paravalvular leakage

**Fig. 1:**
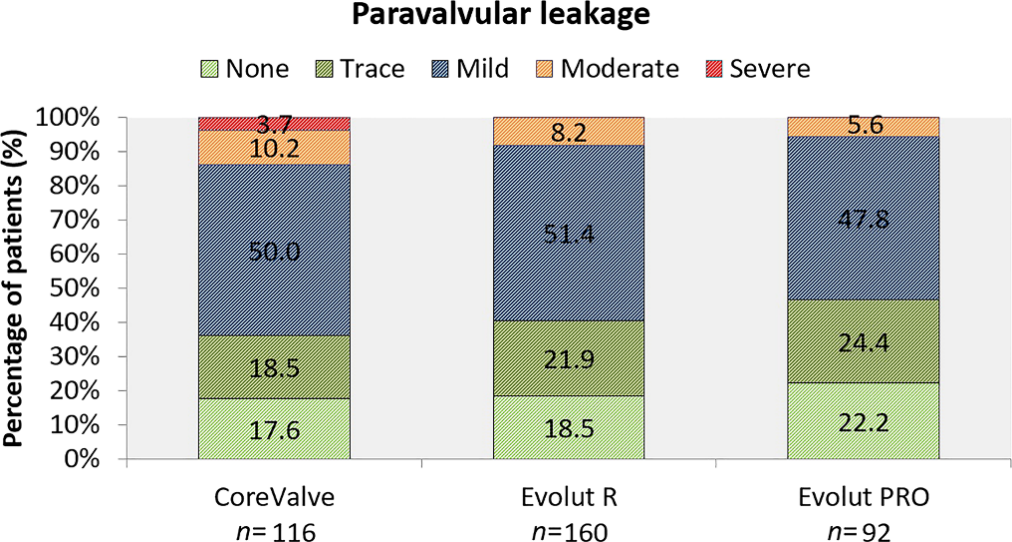
Grading of paravalvular leakage after transcatheter aortic valve implantation

Severe PVL was observed only with CoreValve (4% vs 0% vs 0%, *p* = 0.014), as displayed in Fig. 1. By multivariable regression CoreValve did not show a higher risk than Evolut R for moderate-to-severe PVL (OR 2.06, 95% CI 0.79–5.39, *p* = 0.14). However, CoreValve showed a tendency towards a higher risk for moderate or severe PVL when compared to the combined Evolut platform (OR 2.46, 95% CI 0.98–6.16, *p* = 0.055). Also, male gender and the need for post-dilatation were associated with a higher risk for more-than-mild PVL. Smaller annular sizing ratio (undersizing) and (functional) bicuspid valves were associated with significant PVL in univariate analysis, but not in multivariable analysis. No significant difference in all-cause mortality was observed among the three THVs (7% vs 4% vs 1%, *p* = 0.10, Tab. 2), but the combined Evolut platform showed a tendency towards lower all-cause mortality (7% vs 3%, *p* = 0.06). No significant differences in any stroke or major vascular complications were observed, but there were less major or life-threatening bleedings with Evolut PRO compared to the two other valves (CoreValve 15% vs Evolut R 14% vs Evolut PRO 4%, *p* = 0.034). It is of note that Evolut PRO was associated with persistent (new) RBBB at discharge (0% vs 1% vs 7%, *p* = 0.001).

## Discussion

The main findings of this single-centre comparative study of three consecutive SEVs are:

1. The Evolut platform resulted in a lower PPI rate than CoreValve.
2. Severe PVL occurred only with CoreValve, not with the Evolut platform.
3. The sealing wrap feature of Evolut PRO did not further reduce PVL.

### Need for permanent pacemaker

The lower pacemaker rates of 16% and 18% following TAVI with the Evolut R and Evolut PRO THVs are in line with the currently reported rates, which vary between 15–20% and 12 –19%, respectively [2–5, 9, 10]. The PPI rate was consistently higher with CoreValve, varying between 26% and 29% in high-risk patients [1, 11]. Multivariable regression analysis confirmed the higher PPI rate with CoreValve as compared with Evolut R and PRO. We reported previously that 50% of conduction disorders already started after pre-dilatation and before THV implantation [12]. One could speculate that less pre-dilatation in the Evolut R and PRO cohort could (partially) explain this lower PPI rate. Conversely, post-dilatation was performed more often with the two newer valves, and univariate analysis showed pre-dilatation and post-dilatation not to be significant predictors of PPI. Several design characteristics of the second-generation Evolut R valve may have been clinically relevant [13]. First, the ability to reposition/recapture the device could result in higher implantation depths (although not observed in the present study) [13]. Second, the radial force is distributed more consistently and homogeneously and may create less trauma to the His bundle [13]. Third, its inflow portion was redesigned to deliver a less traumatic implantation and oversizing as well as to achieve a higher implantation depth with a lower risk of embolisation. The Evolut PRO is similar, but comes with an outer porcine pericardial wrap at the lower two rows of the stent frame to minimise PVL [5]. The need for PPI was comparable between patients treated with Evolut R and Evolut PRO, but we found significantly more persistent RBBBs in patients treated with the latter. Hypothetically, the outer pericardial wrap may increase the pressure on the AV node and bundle branches [14]. A recent propensity-matched study comparing transfemoral implantation of Evolut R (*n* = 148) and Evolut PRO (*n* = 74) showed a tendency towards a greater need for PPI after TAVI with the Evolut PRO (10.8% vs 18.6%, *p* = 0.096) [9]. Our study found comparable pacemaker rates in a combined transfemoral and transsubclavian TAVI cohort.

### PVL and other clinical outcomes

The incidence of more-than-mild PVL was higher with CoreValve (13%) than with the Evolut platform (8% and 6%). Our findings are in line with the 8–14% more-than-mild PVL in previous CoreValve reports and lower rates with Evolut R (1–8%) and Evolut PRO (0–6%) [1–5, 9–11]. More-than-mild PVL is associated with higher mortality rates at 1 year [7]. In our study CoreValve showed a tendency towards a higher risk for more-than-mild PVL compared to the Evolut platform, even when corrected for (relative) oversizing and bicuspid anatomy. Also, male gender and need for post-dilatation were associated with moderate-to-severe PVL. Male gender has been associated with more-than-mild PVL in the past, but the underlying mechanism is not completely understood [15]. Males have more extensive aortic root calcification than females, even with similar AS severity [16]. This may explain a higher rate of more-than-mild PVL and was also demonstrated in the SURTAVI trial [15]. Extensive aortic root calcification may inhibit complete valve apposition, induce valve eccentricity with a higher risk for PVL and may require post-dilatation more often [17]. The Evolut PRO features a pericardial wrap to further reduce PVL at the expense of an additional 2‑French in profile (14‑F to 16-F) [9]. This 2‑F larger profile may result in more complications at the access site because there is a direct correlation between SFAR and vascular complications [18]. However, we did not find more vascular complications or bleedings with Evolut PRO than with Evolut R. This may be explained by our growing experience with large-bore access site management. Alternatively, the fact that we reserved the Evolut R predominantly for patients with smaller, more calcified femoral arteries after the commercial launch of Evolut PRO may have hypothetically inflated bleeding and vascular complication rates with Evolut R and skewed the comparison. The overall 30-day all-cause mortality and stroke rates of 4.1% and 3.5% are comparable to rates currently reported with SEVs in higher-risk, elderly patients, varying between 1.7–3.3% and 1.7–4.9%, respectively [1, 4, 5]. There were no significant differences in all-cause mortality or the occurrence of any stroke between the three device generations in our study. However, the combined Evolut (R + PRO) platform experience showed a tendency towards a lower 30-day mortality rate as compared to CoreValve. Intrinsic device iterations but also growing experience, a shift to lower-risk patients and procedure modifications over time may have contributed to the overall safety.

### Study limitations

This was a single-centre, retrospective study and may be subject to inherent bias. The non-randomised, observational nature precludes any definite conclusions. Overall, TAVI experience and changing procedural execution over time cannot be completely corrected through statistical methods. Still, we believe our study population represents a real-world TAVI population and thus enhances generalisability. A low event rate restricted the number of confounders to account for in some analyses. Notably, we observed no significant differences in covariates closely related to PPI or significant PVL. The decision in favour of PPI was at the discretion of the treating physician, but high-degree atrioventricular block was the predominant reason for PPI and is in accordance with ESC guidelines on cardiac pacing. Also, the local policy was to leave a temporary pacemaker in situ less than 24 h to prevent infections. Consequently, if a patient was pacemaker dependent after 24 h, a permanent pacemaker was inserted. This may have prevented conduction disorders from resolving before PPI, but this was standard of care for all SEVs.

## Conclusion

TAVI with SEVs was safe and device iterations may result in a lower need for PPI. More-than-mild PVL seemed to occur less often with repositionable technology.

## Supplementary Information

Supplemental Figure 1 Permanent Pacemaker Implantation

Supplemental Table 1 Univariate logistic regression analysis on need for permanent pacemaker implantation at 30 days after TAVI; Supplemental Table 2 Univariate logistic regression analysis on moderate or severe PVL after TAVI; Supplemental Table 3: Baseline characteristics and procedural details between CoreValve and Evolut platform; Supplemental Table 4: Multivariable regression analysis for need for permanent pacemakers and paravalvular leakage for Evolut R/PRO vs. CoreValve
