# Supplementary figures and images for: Clinical consequences of consecutive self-expanding transcatheter heart valve iterations
Source: Neth Heart J. 2021 Apr 29;30(3):140–8. doi: 10.1007/s12471-021-01568-5 (PMC8881514; doi:10.1007/s12471-021-01568-5)

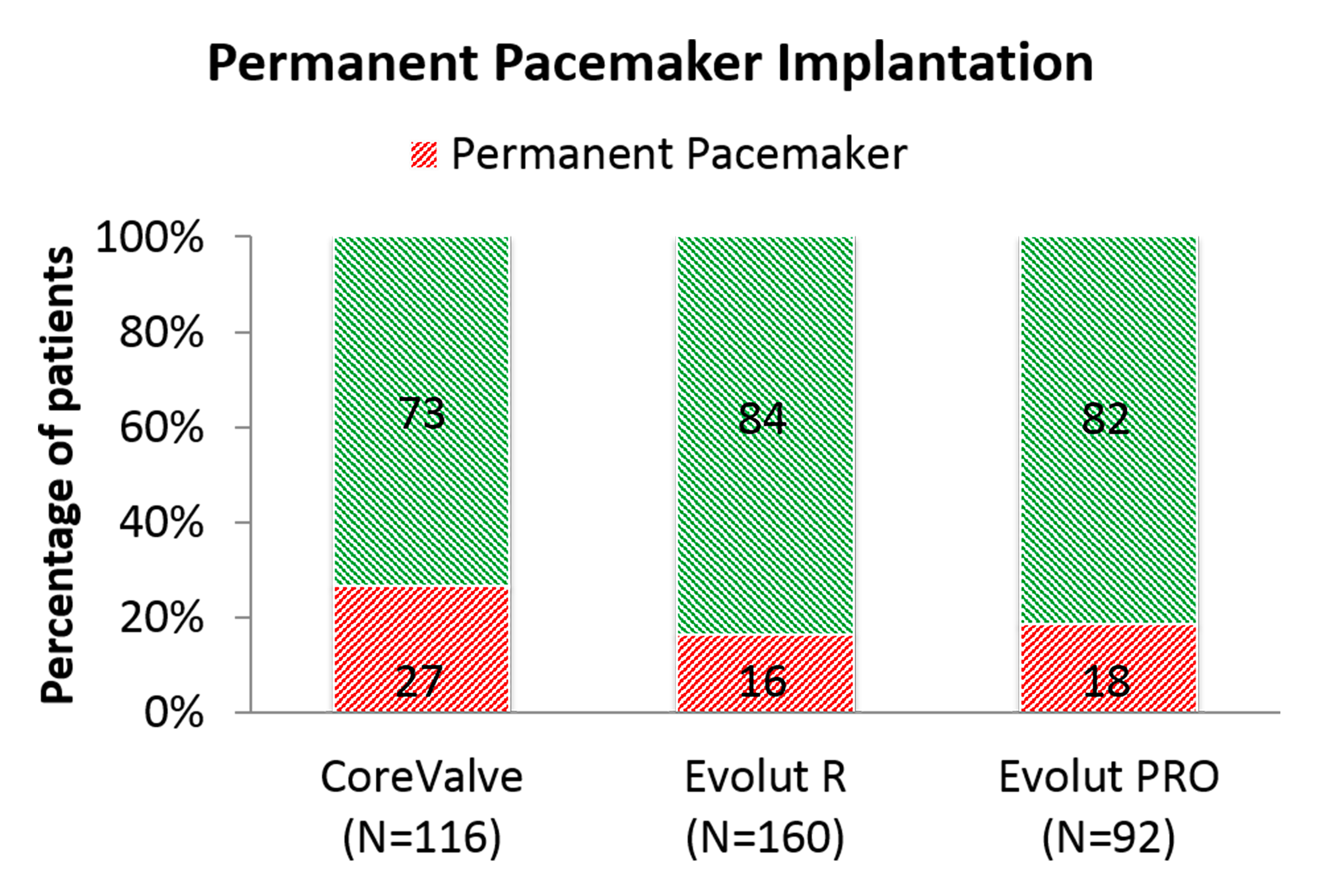

Supplement: Supplementary file 1 — Supplemental Figure 1 Permanent Pacemaker Implantation [file 12471_2021_1568_MOESM1_ESM.jpg]
