# Supplementary material for: Clinical consequences of consecutive self-expanding transcatheter heart valve iterations
Source: Neth Heart J. 2021 Apr 29;30(3):140–8. doi: 10.1007/s12471-021-01568-5 (PMC8881514; doi:10.1007/s12471-021-01568-5)
Supplement: Supplementary file 2 — Supplemental Table 1 Univariate logistic regression analysis on need for permanent pacemaker implantation at 30 days after TAVI; Supplemental Table 2 Univariate logistic regression analysis on moderate or severe PVL after TAVI; Supplemental Table 3: Baseline characteristics and procedural details between CoreValve and Evolut platform; Supplemental Table 4: Multivariable regression analysis for need for permanent pacemakers and paravalvular leakage for Evolut R/PRO vs. CoreValve [file 12471_2021_1568_MOESM2_ESM.docx]

**Table S1: Univariate logistic regression analysis on need for permanent**

**pacemaker implantation at 30 days after TAVI**

|  | **Permanent pacemaker implantation**  OR (95% CI) | **P-value** |
| --- | --- | --- |
| **Baseline characteristics** | | |
| Male gender | 1.694 (1.001-2.865) | **0.049** |
| Age in years | 1.02 (0.99-1.06) | 0.17 |
| STS-score (%) | 1.03 (0.94-1.12) | 0.56 |
| Creatinin on baseline (umol/L) | 1.005 (1.001-1.009) | **0.02** |
| Body mass index (kg/m2) | 1.03 (0.97-1.08) | 0.35 |
| Ischemic Heart Disease | 0.94 (0.56-1.57) | 0.80 |
| History of AVR/TAVI | 0.71 (0.24-2.12) | 0.54 |
| Diabetes mellitus | 0.89 (0.51-1.56) | 0.68 |
| Hypertension | 0.88 (0.49-1.58) | 0.67 |
| History of Atrial Fibrillation | 0.96 (0.53-1.71) | 0.88 |
| History of Stroke | 0.79 (0.37-1.69) | 0.54 |
| Peripheral Artery Disease | 1.59 (0.95-2.66) | 0.075 |
| NYHA class ≥3 | 1.38 (0.78-2.44) | 0.27 |
| Bicuspid valve (functional) | 1.50 (0.60-3.71) | 0.38 |
| Moderate or severe annulus calcification (Rosenhek) | 0.81 (0.41-1.60) | 0.54 |
| Moderate or severe LVOT calcification | 1.06 (0.53-2.15) | 0.87 |
| **Baseline conduction disturbances** | | |
| RBBB | 7.95 (3.73-16.94) | **<0.0005** |
| LBBB | 0.48 (0.16-1.41) | 0.18 |
| UIVD | N/A* | - |
| AV1B | 2.92 (1.66-5.14) | **<0.0005** |
| LAFB | 2.02 (0.91-4.49) | 0.086 |
| LPFB | N/A* | - |
| **Procedural details** | | |
| Evolut R  CoreValve  Evolut PRO | 1.00 (reference)  1.88 (1.04-3.38)  1.17 (0.60-2.29) | **-**  **0.035**  0.65 |
| Femoral access | 0.73 (0.34-1.56) | 0.42 |
| Target access vessel diameter (mm) | 1.04 (0.86-1.26) | 0.70 |
| Sheath to femoral artery ratio | 1.32 (0.37-4.64) | 0.67 |
| Depth of Implantation (mm) | 1.18 (1.08-1.28) | **<0.005** |
| Perimeter derived annular diameter (mm) | 1.17 (1.05-1.29) | **0.004** |
| Annular sizing ratio | 0.03 (0.001-1.128) | 0.058 |
| Pre-dilatation | 1.35 (0.79-2.30) | 0.28 |
| Post-dilatation | 1.11 (0.66-1.87) | 0.70 |
| Repositioning used? | 0.30 (0.12-0.71) | **0.007** |
| Valve in Valve during procedure | 1.14 (0.37-3.58) | 0.82 |

Variables are shown as odds ratio OR (95% confidence interval). Abbreviations: STS = Society of Thoracic Surgeons, AVR = aortic valve replacement, TAVI = transcatheter aortic valve implantation, NYHA = New York Heart Association, LVOT = Left ventricular outflow tract, RBBB = right bundle branch block, LBBB = left bundle branch block, UIVD = unspecific intraventricular conduction defect, AV1B = 1^st^ degree atrioventricular block, LAFB = left anterior fascicular block, LPFB = left posterior fascicular block.

* Not available because of complete separation

**Table S2: Univariate logistic regression analysis on moderate or severe PVL after TAVI**

|  | **Moderate or severe PVL**  OR (95% CI) | **P-value** |
| --- | --- | --- |
| **Baseline characteristics** | | |
| Male gender | 4.25 (1.70-10.61) | **0.002** |
| Age in years | 0.964 (0.927-1.002) | 0.064 |
| STS-score (%) | 0.92 (0.80-1.06) | 0.25 |
| Creatinin on baseline (umol/L) | 1.000 (0.993-1.007) | 0.98 |
| Body mass index (kg/m2) | 0.86 (0.78-0.95) | **0.003** |
| Ischemic Heart Disease | 1.01 (0.49-2.11) | 0.97 |
| History of AVR/TAVI | 0.92 (0.21-4.12) | 0.91 |
| Diabetes mellitus | 0.62 (0.26-1.48) | 0.28 |
| Hypertension | 0.63 (0.29-1.40) | 0.26 |
| History of Atrial Fibrillation | 1.70 (0.80-3.64) | 0.17 |
| History of Stroke | 1.15 (0.42-3.16) | 0.78 |
| Peripheral Artery Disease | 0.78 (0.36-1.66) | 0.51 |
| NYHA class ≥3 | 1.08 (0.49-2.38) | 0.85 |
| Bicuspid valve (functional) | 2.81 (0.97-8.11) | 0.057 |
| Moderate or severe annulus calcification (Rosenhek) | 0.73 (0.28-1.89) | 0.52 |
| Moderate or severe LVOT calcification | 1.01 (0.37-2.79) | 0.98 |
| **Procedural details** | | |
| Evolut R  CoreValve  Evolut PRO | 1.00 (reference)  1.82 (0.82-4.07)  0.66 (0.22-1.93) | **-**  0.14  0.45 |
| Femoral access | 0.96 (0.27-3.34) | 0.94 |
| Target access vessel diameter (mm) | 1.28 (0.95-1.72) | 0.11 |
| Sheath to femoral artery ratio | 0.51 (0.06-4.12) | 0.53 |
| Depth of Implantation (mm) | 1.06 (0.94-1.19) | 0.38 |
| Perimeter derived annular diameter (mm) | 1.31 (1.13-1.51) | **<0.005** |
| Annular sizing ratio | 0.002 (0.000-0.203) | **0.009** |
| Pre-dilatation | 1.39 (0.65-2.95) | 0.40 |
| Post-dilatation | 3.75 (1.74-8.06) | **0.001** |
| Repositioning used? | 0.78 (0.29-2.13) | 0.63 |
| Valve in Valve during procedure | 0.64 (0.08-4.98) | 0.67 |

Variables are shown as odds ratio OR (95% confidence interval). Abbreviations: STS = Society of Thoracic Surgeons, AVR = aortic valve replacement, TAVI = transcatheter aortic valve implantation, NYHA = New York Heart Association, LVOT = Left ventricular outflow tract.

**Table S3: Baseline characteristics and procedural details between CoreValve and Evolut platform**

|  | **CoreValve**  **(N = 116)** | | **Evolut R/PRO**  **(N = 252)** | **P-value** |
| --- | --- | --- | --- | --- |
| **Baseline Characteristics** | | | | |
| Male gender | 68 (59%) | | 128 (51%) | 0.16 |
| Age in years | 80 [75-84] | | 80 [73-85] | 0.93 |
| STS-score (%) | 4.3 [3.1-5.7] | | 4.2 [2.6-6.4] | 0.61 |
| Creatinin on baseline (umol/L) | 95 [75-128] | | 94 [75-117] | 0.45 |
| Body mass index (kg/m2) | 27±4 | | 27±5 | 0.87 |
| Ischemic Heart Disease | 53 (46%) | | 101 (40%) | 0.31 |
| History of AVR/TAVI | 5 (4%) | | 21 (8%) | 0.16 |
| Diabetes mellitus | 38 (33%) | | 73 (29%) | 0.49 |
| Hypertension | 87 (75%) | | 192 (76%) | 0.71 |
| History of Atrial Fibrillation | 28 (24%) | | 69 (27%) | 0.51 |
| History of Stroke | 21 (18%) | | 32 (13%) | 0.17 |
| Peripheral Arterial Disease | 43 (37%) | | 107 (42%) | 0.31 |
| NYHA class ≥3 | 84 (75%) | | 155 (62%) | **0.026** |
| Bicuspid valve (functional) | 9 (8%) | | 17 (7%) | 0.79 |
| Moderate or severe annulus calcification (Rosenhek) | 92 (79%) | | 203 (81%) | 0.62 |
| Moderate or severe LVOT calcification | 20 (17%) | | 36 (14%) | 0.75 |
| **Baseline conduction disturbances (alone or in combination)** | | | | |
| RBBB | 7 (6%) | | 26 (10%) | 0.18 |
| LBBB | 18 (16%) | | 17 (7%) | **0.008** |
| UIVD | 3 (3%) | | 6 (2%) | 0.92 |
| AV1B | 26 (22%) | | 49 (19%) | 0.54 |
| LAFB | 15 (13%) | | 165 (6%) | **0.037** |
| LPFB | 1 (1%) | | 1 (<1%) | 0.53 |
| **Procedural details** | | | | |
| Femoral access | 97 (84%) | | 231 (92%) | **0.021** |
| Target access vessel diameter (mm) | 7.0±1.6 | | 7.0±1.4 | 0.72 |
| Sheath to femoral artery ratio | 1.03 [0.87-1.23] | | 0.85 [0.74-0.95] | **<0.005** |
| Perimeter derived annular diameter (mm) | 25.0 [23.4-26.7] | | 23.9 [22.4-25.2] | **<0.005** |
| Annular sizing ratio | 1.15 [1.10-1.20] | | 1.18 [1.15-1.23] | **<0.005** |
| Depth of Implantation (mm) | 7.0±3.2 | | 7.1±3.0 | 0.82 |
| Pre-dilatation | 92 (79%) | | 23 (9%) | **<0.005** |
| Post-dilatation | 32 (28%) | | 105 (42%) | **0.009** |
| Repositioning used? | - | | 73 (30%) | **<0.005** |
| Valve in Valve during procedure | 8 (7%) | | 10 (4%) | 0.23 |
| Number of valves implanted | 1, range 1-3 | | 1, range 1-3 | 0.06 |
| **Clinical outcomes** | | | | |
| Permanent pacemaker post TAVI owing to: | | 31 (27%) | 43 (17%) | **0.032** |
| AV3B | | 20 (65%) | 37 (86%) |  |
| AV2B (Mobitz II) | | 3 (10%) | 4 (9%) |  |
| AF with bradycardia | | 3 (10%) | 0 (0%) |  |
| Sick sinus syndrome | | 3 (10%) | 0 (0%) |  |
| Other | | 2 (7%) | 2 (5%) |  |
| Mortality at 30 days | | 8 (7%) | 7 (3%) | 0.06 |
| Moderate to severe paravalvular leakage  Moderate  Severe | | 15 (13%)  11 (10%)  4 (3%) | 17 (7%)  17 (7%)  0 (0%) | **0.044** |
| Minor vascular complication | | 11 (10%) | 14 (6%) | 0.16 |
| Major vascular complication | | 12 (10%) | 23 (9%) | 0.71 |
| Minor bleeding | | 7 (6%) | 20 (8%) | 0.52 |
| Major or life-threatening bleeding  Major  Life-threatening | | 17 (15%)  8 (7%)  9 (8%) | 27 (11%)  17 (7%)  10 (4%) | 0.28 |
| Any stroke  Disabling | | 7 (6%)  5 (4%) | 6 (2%)  3 (1%) | 0.12 |
| **New conduction disturbances** | | | | |
| Procedural AV3B | | 24 (21%) | 44 (17%) | 0.49 |
| Procedural LBBB | | 65 (56%) | 138 (55%) | 0.88 |
| Procedural RBBB | | 2 (2%) | 2 (1%) | 0.59 |
| Temporary LBBB | | 37 (32%) | 70 (28%) | 0.42 |
| Permanent LBBB | | 35 (30%) | 80 (32%) | 0.77 |
| Temporary RBBB | | 4 (4%) | 12 (5%) | 0.58 |
| Permanent RBBB | | 0 (0%) | 7 (3%) | 0.10 |
| Temporary AV2B/AV3B | | 8 (7%) | 23 (9%) | 0.46 |
| Permanent AV2B/AV3B | | 20 (17%) | 31 (12%) | 0.21 |
| AV1B | | 22 (19%) | 44 (17%) | 0.74 |
| AF | | 16 (14%) | 11 (4%) | **0.001** |

Categorical variables are shown as n (%). Continuous variables are displayed as mean±SD, median [interquartile range] or median, range. Abbreviations: STS = Society of Thoracic Surgeons, AVR = aortic valve replacement, TAVI = transcatheter aortic valve implantation, NYHA = New York Heart Association, RBBB = right bundle branch block, LBBB = left bundle branch block, UIVD = unspecific intraventricular conduction defect, AV1B = 1^st^ degree atrioventricular block, LAFB = left anterior fascicular block, LPFB = left posterior fascicular block, AV3B = 3^rd^ degree atrioventricular block, AV2B = 2^nd^ degree atrioventricular block, AF = Atrial fibrillation.

**Table S4: Multivariable regression analysis for need for permanent pacemakers and paravalvular leakage for Evolut R/PRO vs. CoreValve**

|  | **Permanent pacemaker implantation**  OR (95% CI) | **P-value** |
| --- | --- | --- |
| Type of THV used  Medtronic Evolut R/PRO  Medtronic CoreValve | 1.00 (reference)  2.56 (1.29-5.08) | **-**  **0.007** |
| Male gender | 0.61 (0.30-1.25) | 0.18 |
| RBBB at baseline | 15.00 (5.71-39.43) | **<0.005** |
| LAFB at baseline | 1.27 (0.46-3.52) | 0.65 |
| AV1B at baseline | 3.78 (1.83-7.79) | **<0.005** |
| Mean depth of implantation (mm) | 1.22 (1.09-1.36) | **<0.005** |
| Annular sizing ratio | 0.099 (0.001-7.138) | 0.29 |
| Creatinine at baseline (umol/L) | 1.002 (0.998-1.007) | 0.27 |
| Peripheral artery disease | 1.59 (0.84-2.99) | 0.15 |
|  | **Moderate or severe PVL**  OR (95% CI) | **P-value** |
| THV-type  Medtronic Evolut R/PRO  Medtronic CoreValve | 1.00 (reference)  2.46 (0.98-6.16) | **-**  0.055 |
| Bicuspid valve (functional) | 1.21 (0.35-4.13) | 0.76 |
| Annular sizing ratio | 0.096 (0.000-26.722) | 0.41 |
| Post-dilatation | 5.75 (2.26-14.58) | **<0.005** |
| Male gender | 5.47 (1.79-16.65) | **0.003** |

Variables are shown as odds ratio OR (95% confidence interval). Abbreviations: THV = Transcatheter Heart Valve; RBBB = Right Bundle Branch Block; LAFB = Left Anterior Fascicular Block; AV1B = 1^st^ degree Atrioventricular Block.
